# Supplementary material for: Variants in MHY7 Gene Cause Arrhythmogenic Cardiomyopathy
Source: Genes (Basel). 2021 May 22;12(6):793. doi: 10.3390/genes12060793 (PMC8224781; doi:10.3390/genes12060793)
Supplement: Supplementary file 1 [file genes-12-00793-s001.zip › genes-1228828-supplementary.pdf]

Supplementary Table 1: Genes studied for ACM

**Gene**	**RefSeq**
DSC2	NM_024422.6
DSG2	NM_001943.5
DSP	NM_004415.4
JUP	NM_002230.4
PKP2	NM_001005242.3

Supplementary Table 2: Bioinformatics score of M877T variant

	**Wild-type**	**M877T mutant**
HADDOCK score	-322.1 +/- 7.7	-311.7 +/- 6.8
Cluster size	30	26
RMSD from the overall lowest-energy structure	13.8 +/- 0.7	3.5 +/- 0.4
Van der Waals energy	-133.5 +/- 12.5	-142.7 +/- 15.5
Electrostatic energy	-808.8 +/- 25.1	-862.7 +/- 75.5
Desolvation energy	-219.7 +/- 19.1	-189.1 +/- 16.8
Restraints violation energy	1927.7 +/- 100.66	1926.1 +/- 178.41
Buried Surface Area	6286.9 +/- 244.4	6290.5 +/- 62.0
